# Characteristics and Trends of the Most Cited Publications in *The Journal of Arthroplasty*

**DOI:** 10.1016/j.artd.2022.05.011

**Published:** 2022-07-19

**Authors:** Dylan Luxenburg, David Constantinescu, Gemma St. Louis, Kevin J. Bondar, Suleiman Y. Sudah, Michele D’Apuzzo

**Affiliations:** a University of Miami Leonard M. Miller School of Medicine, Miami, FL, USA; b Department of Orthopaedic Surgery, University of Miami Hospital, Miami, FL, USA; c Department of Orthopedic Surgery, Monmouth Medical Center, Long Branch, NJ, USA

**Keywords:** Citation analysis, *The Journal of Arthroplasty*, Publications, Bibliometric study, Top-cited

## Abstract

**Background:**

This study aims to identify the most frequently cited articles published in the *Journal of Arthroplasty* (*JOA*) and to analyze the trends in the content and contributors of the literature within the journal.

**Methods:**

The 100 most cited articles published in the *JOA* were accessed using the Scopus database. The number of citations, year of publication, level of evidence (LOE), article type, country of origin, and contributing institution were each recorded for each article.

**Results:**

The United States (63%) was the most prolific publishing nation. The 1990s (30%) and 2000s (47%) were the most productive decades. The most common article category was clinical outcomes (33%), followed by technical note (16%) and biomechanics (14%). The plurality of the top 100 articles were well-designed case-control or cohort studies of LOE II (46%) followed by LOE V (32%) and LOE I (11%).

**Conclusions:**

Using citation analysis, the most influential articles in the *JOA* were comprehensively and objectively analyzed. The most popular fields of research involved clinical outcomes (33%) and technical note (16%), both of which increase an article’s likelihood of being highly cited. Knowledge of the most influential articles in the *JOA* allows for appreciation of current and potential future areas of literature regarding diagnosis, management, and outcome of a patient undergoing arthroplasty.

## Introduction

Peer-reviewed literature remains the foundation of how evidence-based medicine is practiced by clinicians all around the world. Total joint arthroplasty (TJA), including both total hip arthroplasty (THA) and total knee arthroplasty, comprises a large cohort of surgical procedures performed in the United States of America (USA) [1]. The ever-growing body of literature across numerous journals regarding arthroplasty provides a challenge to sift through and find the highest quality of evidence studies that can directly impact patient care.

Bibliometric analysis has become a popular method to identify and analyze specific topics or trends by using the most influential articles in each topic or journal. The analysis is run by order of citations to determine the impact and influence of each article. This method has been used to highlight topics of interest, shed light on under-researched topics, and determine the quality of contributions from various orthopaedic journals [2,3]. This method has also been useful for analyzing orthopaedic injuries and procedures such as meniscal injuries, unicondylar knee arthroplasty, and hip and knee arthroplasty [[4], [5], [6]].

*The Journal of Arthroplasty* (*JOA*) is one of the top journals in orthopaedic surgery and the number 1 journal focusing on joint arthroplasty of the hip and knee when measured by impact factor. There is, however, lack of a coherent summary of the most relevant content of this journal. Our study aims to analyze the characteristics and trends of the top 100 most cited articles in the *JOA*. The purpose of this analysis is to elucidate the influence this journal has had in its various areas of research focus. We hypothesize that most of the literature comes from research groups out of the USA and are focused on clinical outcomes regarding prosthetic joint infection.

## Material and methods

In September 2021, the Scopus database was used to identify articles published in the *JOA*. Preferred Reporting Items for Systematic Reviews and Meta-Analyses guidelines were used in the collection of data. We began by defining the topic of this study as a bibliometric analysis of articles published in the *JOA*. Our search term in the Scopus database was “Arthroplasty,” and our “search within” was set to “source title.” We searched the journal since its inception in 1986 for all studies it has published. The articles were sorted in descending order of times cited. All included studies were written in English and peer reviewed.

A combination of Scopus metrics and data collection via author review was performed using Microsoft Excel. The following data were extracted from each article: title, study design, main topic, citations, year, institutional affiliation, country of origin, and level of evidence (LOE). The countries of origin were determined by the locations of the authors’ affiliated institutions. The order of the top 100 articles was determined by the number of citations per each article. If multiple articles had the same number of citations, then the most recently published article had a prioritized ranking. The LOE was either provided upon evaluation of the abstract or determined by full-text review using the Oxford LOE Guidelines. The lead author (DL) determined article classifications of each study which included the following: surgical technique, clinical outcomes, anatomy/biomechanical, natural history, clinical guidelines, classification, imaging, technical note, and biomechanics. The following topics were used to classify publications: primary hip arthroplasty, primary knee arthroplasty, prosthetic joint infection, hip and knee arthroplasty, revision hip arthroplasty, primary shoulder arthroplasty, healthy knee, venous thromboembolism, implant, and revision knee arthroplasty. The topic of TJA was applied to articles that evaluated joint arthroplasty in more than 2 joints. Articles were placed into only one category via reviewer determination, and this single most applicable category for that article was selected. These data were verified by a separate investigator, with discrepancies being reviewed by the lead author.

## Results

**Figure 1:**
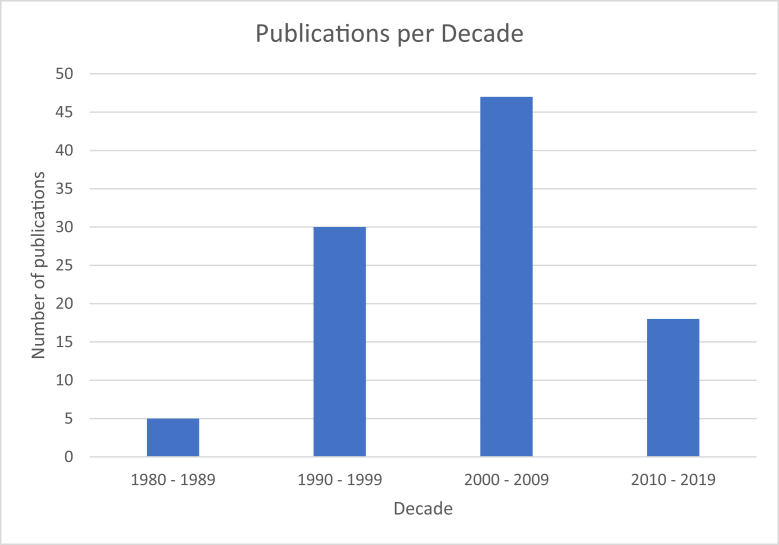
Top 100 articles in the *Journal of Arthroplasty* by decade.

The top 100 most cited articles meeting our inclusion criteria were analyzed. All articles were published between 1987 and 2018. The greatest number of citations in 1 article was 914, while the least number was 169 (Table 1). The total amount of citations was 26,278, which provides an average of 263 citations per article. In terms of productivity by decade, the 2000s were the most productive (n = 47 publications), followed by the 1990s (n = 30 publications), the 2010s (n = 18 publications), and lastly the 1980s (n = 5 publications) (Fig. 1).

**Table 1:** The top 100 most cited articles.

Rank	Publication	Total citation
1	Kurtz S.M., Lau E., Watson H., Schmier J.K., Parvizi J. Economic burden of periprosthetic joint infection in the united states. Journal of Arthroplasty. 2012	914
2	Paprosky W.G., Perona P.G., Lawrence J.M. Acetabular defect classification and surgical reconstruction in revision arthroplasty. A 6-year follow-up evaluation. The Journal of Arthroplasty. 1994	697
3	Kurtz S.M., Lau E., Schmier J., Ong K.L., Zhao K., Parvizi J. Infection Burden for Hip and Knee Arthroplasty in the United States. Journal of Arthroplasty. 2008	662
4	Walch G., Badet R., Boulahia A., Khoury A. Morphologic study of the glenoid in primary glenohumeral osteoarthritis. Journal of Arthroplasty. 1999	631
5	Zahiri C.A., Schmalzried T.P., Szuszczewicz E.S., Amstutz H.C. Assessing activity in joint replacement patients. Journal of Arthroplasty. 1998	579
6	[No author name available] Oral Thrombin Inhibitor Dabigatran Etexilate vs North American Enoxaparin Regimen for Prevention of Venous Thromboembolism After Knee Arthroplasty Surgery. Journal of Arthroplasty. 2009	543
7	Muratoglu O.K., Bragdon C.R., O'Connor D.O., Jasty M., Harris W.H. A novel method of cross-linking ultra-high-molecular-weight polyethylene to improve wear, reduce oxidation, and retain mechanical properties: Recipient of the 1999 HAP Paul award. Journal of Arthroplasty. 2001	508
8	Dumbleton J.H., Manley M.T., Edidin A.A. A literature review of the association between wear rate and osteolysis in total hip arthroplasty. Journal of Arthroplasty. 2002	475
9	Franklin J.L., Barrett W.P., Matsen F.A., III Glenoid loosening in total shoulder arthroplasty: Association with rotator cuff deficiency. Journal of Arthroplasty. 1988	458
10	Kennedy J.G., Rogers W.B., Soffe K.E., Sullivan R.J., Griffen D.G., Sheehan L.J. Effect of acetabular component orientation on recurrent dislocation, pelvic osteolysis, polyethylene wear, and component migration. Journal of Arthroplasty. 1998	425
11	Massin P., Engh C.A. Evaluation of cementless acetabular component migration: An experimental study. Journal of Arthroplasty. 1989	409
12	Parvizi J., Tan T.L., Goswami K., Higuera C., Della Valle C., Chen A.F., Shohat N. The 2018 Definition of Periprosthetic Hip and Knee Infection: An Evidence-Based and Validated Criteria. Journal of Arthroplasty. 2018	397
13	Parvizi J., Gehrke T. Definition of periprosthetic joint infection. Journal of Arthroplasty. 2014	393
14	Jolles B.M., Zangger P., Leyvraz P.-F. Factors predisposing to dislocation after primary total hip arthroplasty: A multivariate analysis. Journal of Arthroplasty. 2002	382
15	Fang D.M., Ritter M.A., Davis K.E. Coronal Alignment in Total Knee Arthroplasty. Just How Important is it? Journal of Arthroplasty. 2009	369
16	Mason J.B., Fehring T.K., Estok R., Banel D., Fahrbach K. Meta-Analysis of Alignment Outcomes in Computer-Assisted Total Knee Arthroplasty Surgery. Journal of Arthroplasty. 2007	368
17	Sharkey P.F., Lichstein P.M., Shen C., Tokarski A.T., Parvizi J. Why are total knee arthroplasties failing today-has anything changed after 10 years? Journal of Arthroplasty. 2013	358
18	Namba R.S., Paxton L., Fithian D.C., Stone M.L. Obesity and perioperative morbidity in total hip and total knee arthroplasty patients. Journal of Arthroplasty. 2005	343
19	Ong K.L., Kurtz S.M., Lau E., Bozic K.J., Berry D.J., Parvizi J. Prosthetic Joint Infection Risk After Total Hip Arthroplasty in the Medicare Population. Journal of Arthroplasty. 2009	323
20	Choong P.F., Dowsey M.M., Stoney J.D. Does Accurate Anatomical Alignment Result in Better Function and Quality of Life? Comparing Conventional and Computer-Assisted Total Knee Arthroplasty. Journal of Arthroplasty 2009	305
21	Kienapfel H., Sprey C., Wilke A., Griss P. Implant fixation by bone ingrowth. Journal of Arthroplasty. 1999	297
22	Price A.J., Webb J., Topf H., Dodd C.A.F., Goodfellow J.W., Murray D.W. Rapid recovery after Oxford unicompartmental arthroplasty through a short incision. Journal of Arthroplasty. 2001	287
23	Lindahl H., Malchau H., Herberts P., Garellick G. Periprosthetic femoral fractures: Classification and demographics of 1049 periprosthetic femoral fractures from the Swedish National Hip Arthroplasty Register. Journal of Arthroplasty. 2005	287
24	Mancuso C.A., Salvati E.A., Johanson N.A., Peterson M.G.E., Charlson M.E. Patients' expectations and satisfaction with total hip arthroplasty. Journal of Arthroplasty. 1997	279
25	Behrend H., Giesinger K., Giesinger J.M., Kuster M.S. The “Forgotten Joint” as the Ultimate Goal in Joint Arthroplasty. Validation of a New Patient-Reported Outcome Measure. Journal of Arthroplasty. 2012	276
26	Kwon Y.-M., Ostlere S.J., McLardy-Smith P., Athanasou N.A., Gill H.S., Murray D.W. “Asymptomatic” Pseudotumors After Metal-on-Metal Hip Resurfacing Arthroplasty. Prevalence and Metal Ion Study. Journal of Arthroplasty. 2011	267
27	Barrett W.P., Turner S.E., Leopold J.P. Prospective randomized study of direct anterior vs postero-lateral approach for total hip arthroplasty. Journal of Arthroplasty. 2013	266
28	Bullens P.H.J., Van Loon C.J.M., De Waal Malefijt M.C., Laan R.F.J.M., Veth R.P.H. Patient satisfaction after total knee arthroplasty: A comparison between subjective and objective outcome assessments. Journal of Arthroplasty. 2001	265
29	Masri B.A., Duncan C.P., Beauchamp C.P. Long-term elution of antibiotics from bone-cement: An in vivo study using the prosthesis of antibiotic-loaded acrylic cement (PROSTALAC) system. Journal of Arthroplasty. 1998	259
30	Bozic K.J., Chan V., Valone F.H., Feeley B.T., Vail T.P. Trends in hip arthroscopy utilization in the United States. Journal of Arthroplasty. 2013	258
31	Parvataneni H.K., Shah V.P., Howard H., Cole N., Ranawat A.S., Ranawat C.S. Controlling Pain After Total Hip and Knee Arthroplasty Using a Multimodal Protocol With Local Periarticular Injections. A Prospective Randomized Study. Journal of Arthroplasty. 2007	253
32	Malinzak R.A., Ritter M.A., Berend M.E., Meding J.B., Olberding E.M., Davis K.E. Morbidly Obese, Diabetic, Younger, and Unilateral Joint Arthroplasty Patients Have Elevated Total Joint Arthroplasty Infection Rates. Journal of Arthroplasty. 2009	252
33	Burroughs B.R., Hallstrom B., Golladay G.J., Hoeffel D., Harris W.H. Range of motion and stability in total hip arthroplasty with 28-, 32-, 38-, and 44-mm femoral head sizes: An in vitro study. Journal of Arthroplasty. 2005	246
34	Longstaff L.M., Sloan K., Stamp N., Scaddan M., Beaver R. Good Alignment After Total Knee Arthroplasty Leads to Faster Rehabilitation and Better Function. Journal of Arthroplasty. 2009	246
35	Banks S.A., Markovich G.D., Hodge W.A. In vivo kinematics of cruciate-retaining and -substituting knee arthroplasties. Journal of Arthroplasty. 1997	243
36	Chelly J.E., Greger J., Gebhard R., Coupe K., Clyburn T.A., Buckle R., Criswell A. Continuous femoral blocks improve recovery and outcome of patients undergoing total knee arthroplasty. Journal of Arthroplasty. 2001	243
37	Brady O.H., Garbuz D.S., Masri B.A., Duncan C.P. The reliability and validity of the Vancouver classification of femoral fractures after hip replacement. Journal of Arthroplasty. 2000	239
38	Bobyn J.D., Toh K.-K., Hacking S.A., Tanzer M., Krygier J.J. Tissue response to porous tantalum acetabular cups: A canine model. Journal of Arthroplasty. 1999	236
39	Cartier P., Sanouiller J.-L., Grelsamer R.P. Unicompartmental knee arthroplasty surgery: 10-year minimum follow-up period. Journal of Arthroplasty. 1996	234
40	DiGioia III A.M., Plakseychuk A.Y., Levison T.J., Jaramaz B. Mini-incision technique for total hip arthroplasty with navigation. Journal of Arthroplasty. 2003	234
41	Penner M.J., Masri B.A., Duncan C.P. Elution characteristics of vancomycin and tobrarnycin combined in acrylic bone-cement. Journal of Arthroplasty. 1996	233
42	Collier J.P., Sperling D.K., Currier J.H., Sutula L.C., Saum K.A., Mayor M.B. Impact of gamma sterilization on clinical performance of polyethylene in the knee. Journal of Arthroplasty. 1996	233
43	Oral E., Christensen S.D., Malhi A.S., Wannomae K.K., Muratoglu O.K. Wear Resistance and Mechanical Properties of Highly Cross-linked, Ultrahigh-Molecular Weight Polyethylene Doped With Vitamin E. Journal of Arthroplasty. 2006	233
44	Anderson J.G., Wixson R.L., Tsai D., Stulberg S.D., Chang R.W. Functional outcome and patient satisfaction in total knee patients over the age of 75. Journal of Arthroplasty. 1996	231
45	Walter W.L., O'Toole G.C., Walter W.K., Ellis A., Zicat B.A. Squeaking in Ceramic-on-Ceramic Hips. The Importance of Acetabular Component Orientation. Journal of Arthroplasty. 2007	231
46	DiGioia A.M., III, Jaramaz B., Plakseychuk A.Y., Moody J.E., Jr., Nikou C., LaBarca R.S., Levison T.J., Picard F.Comparison of a mechanical acetabular alignment guide with computer placement of the socket. Journal of Arthroplasty. 2002	230
47	Schroer W.C., Berend K.R., Lombardi A.V., Barnes C.L., Bolognesi M.P., Berend M.E., Ritter M.A., Nunley R.M. Why are total knees failing today? Etiology of total knee revision in 2010 and 2011. Journal of Arthroplasty. 2013	230
48	Nevelos J., Ingham E., Doyle C., Streicher R., Nevelos A., Walter W., Fisher J. Microseparation of the centers of alumina-alumina artificial hip joints during simulator testing produces clinically relevant wear rates and patterns. Journal of Arthroplasty. 2000	229
49	Lynch A.F., Rorabeck C.H., Bourne R.B. Extensor mechanism complications following total knee arthroplasty. Journal of Arthroplasty. 1987	224
50	Widmer K.-H. A simplified method to determine acetabular cup anteversion from plain radiographs. Journal of Arthroplasty. 2004	224
51	Decking R., Markmann Y., Fuchs J., Puhl W., Scharf H.-P. Leg axis after computer-navigated total knee arthroplasty: A prospective randomized trial comparing computer-navigated and manual implantation. Journal of Arthroplasty. 2005	224
52	Eldridge J.D.J., Smith E.J., Hubble M.J., Whitehouse S.L., Learmonth I.D. Massive early subsidence following femoral impaction grafting. Journal of Arthroplasty. 1997	219
53	Griffin F.M., Insall J.N., Scuderi G.R. Accuracy of soft tissue balancing in total knee arthroplasty. Journal of Arthroplasty. 2000	216
54	Dennis D.A., Komistek R.D., Stiehl J.B., Walker S.A., Dennis K.N. Range of motion after total knee arthroplasty: The effect of implant design and weight-bearing conditions. Journal of Arthroplasty. 1998	213
55	Sadoghi P., Liebensteiner M., Agreiter M., Leithner A., Böhler N., Labek G. Revision surgery after total joint arthroplasty: A complication-based analysis using worldwide arthroplasty registers. Journal of Arthroplasty. 2013	213
56	Mizner R.L., Petterson S.C., Clements K.E., Zeni J.A., Irrgang J.J., Snyder-Mackler L. Measuring Functional Improvement After Total Knee Arthroplasty Requires Both Performance-Based and Patient-Report Assessments. A Longitudinal Analysis of Outcomes. Journal of Arthroplasty. 2011	212
57	Devane P.A., Horne J.G., Martin K., Coldham G., Krause B. Three-dimensional polyethylene wear of a press-fit titanium prosthesis: Factors influencing generation of polyethylene debris. Journal of Arthroplasty. 1997	209
58	Dowson D., Hardaker C., Flett M., Isaac G.H. A hip joint simulator study of the performance of metal-on-metal joints: Part II: Design. Journal of Arthroplasty. 2004	208
59	Landy M.M., Walker P.S. Wear of ultra-high-molecular-weight polyethylene components of 90 retrieved knee prostheses. Journal of Arthroplasty. 1988	204
60	Hetaimish B.M., Khan M.M., Simunovic N., Al-Harbi H.H., Bhandari M., Zalzal P.K. Meta-Analysis of Navigation vs Conventional Total Knee Arthroplasty. Journal of Arthroplasty. 2012	202
61	Petersen T.L., Engh G.A.Radiographic assessment of knee alignment after total knee arthroplasty. Journal of Arthroplasty. 1988	199
62	Reuben J.D., Meyers S.J., Cox D.D., Elliott M., Watson M., Shim S.D. Cost comparison between bilateral simultaneous, staged, and unilateral total joint arthroplasty. Journal of Arthroplasty. 1998	197
63	Parvizi J., Pawasarat I.M., Azzam K.A., Joshi A., Hansen E.N., Bozic K.J. Periprosthetic joint infection: The economic impact of methicillin-resistant infections. Journal of Arthroplasty. 2010	197
64	Asayama I., Chamnongkich S., Simpson K.J., Kinsey T.L., Mahoney O.M. Reconstructed hip joint position and abductor muscle strength after total hip arthroplasty. Journal of Arthroplasty. 2005	196
65	Chimento G.F., Pavone V., Sharrock N., Kahn B., Cahill J., Sculco T.P. Minimally invasive total hip arthroplasty: A prospective randomized study. Journal of Arthroplasty. 2005	194
66	Dorr L.D., Kane III T.J., Conaty J.P. Long-term results of cemented total hip arthroplasty in patients 45 years old or younger. A 16-year follow-up study. The Journal of Arthroplasty. 1994	192
67	Anderson K.C., Buehler K.C., Markel D.C. Computer assisted navigation in total knee arthroplasty: Comparison with conventional methods. Journal of Arthroplasty. 2005	192
68	Mahaluxmivala J., Bankes M.J.K., Nicolai P., Aldam C.H., Allen P.W. The effect of surgeon experience on component positioning in 673 press fit condylar posterior cruciate-sacrificing total knee arthroplasties. Journal of Arthroplasty. 2001	188
69	Chin P.L., Kuang Y.Y., Seng J.Y., Ngai N.L. Randomized control trial comparing radiographic total knee arthroplasty implant placement using computer navigation versus conventional technique. Journal of Arthroplasty. 2005	187
70	Brand R.A., Pedersen D.R., Davy D.T., Kotzar G.M., Heiple K.G., Goldberg V.M. Comparison of hip force calculations and measurements in the same patient. The Journal of Arthroplasty. 1994	185
71	Griffin F.M., Math K., Scuderi G.R., Insall J.N., Poilvache P.L. Anatomy of the epicondyles of the distal femur: MRI analysis of normal knees. Journal of Arthroplasty. 2000	184
72	Boyd A.D., Jr., Thomas W.H., Scott R.D., Sledge C.B., Thornhill T.S. Total shoulder arthroplasty versus hemiarthroplasty: Indications for glenoid resurfacing. Journal of Arthroplasty. 1990	183
73	Walter W.L., Insley G.M., Walter W.K., Tuke M.A. Edge loading in third generation alumina ceramic-on-ceramic bearings: Stripe wear. Journal of Arthroplasty. 2004	179
74	Kop A.M., Swarts E. Corrosion of a Hip Stem With a Modular Neck Taper Junction. A Retrieval Study of 16 Cases. Journal of Arthroplasty. 2009	179
75	Restrepo C., Parvizi J., Pour A.E., Hozack W.J. Prospective Randomized Study of Two Surgical Approaches for Total Hip Arthroplasty. Journal of Arthroplasty. 2010	178
76	Iorio R., Clair A.J., Inneh I.A., Slover J.D., Bosco J.A., Zuckerman J.D. Early Results of Medicare's Bundled Payment Initiative for a 90-Day Total Joint Arthroplasty Episode of Care. Journal of Arthroplasty. 2016	178
77	Oswald M.H., Schneider E. Radiological analysis of normal axial alignment of femur and tibia in view of total knee arthroplasty. Journal of Arthroplasty. 1993	177
78	Muratoglu O.K., Bragdon C.R., O'Connor D., Perinchief R.S., Estok II D.M., Jasty M., Harris W.H. Larger diameter femoral heads used in conjunction with a highly cross-linked ultra-high molecular weight polyethylene: A new concept. Journal of Arthroplasty. 2001	176
79	Schmalzried T.P., Peters P.C., Maurer B.T., Bragdon C.R., Harris W.H. Long-duration metal-on-metal total hip arthroplasties with low wear of the articulating surfaces. Journal of Arthroplasty. 1996	175
80	Blunn G.W., Joshi A.B., Minns R.J., Lidgren L., Lilley P., Ryd L., Engelbrecht E., Walker P.S. Wear in retrieved condylar knee arthroplasties: A comparison of wear in different designs of 280 retrieved condylar knee prostheses. Journal of Arthroplasty. 1997	175
81	Edidin A.A., Pruitt L., Jewett C.W., Crane D.J., Roberts D., Kurtz S.M. Plasticity-induced damage layer is a precursor to wear in radiation- cross-linked UHMWPE acetabular components for total hip replacement. Journal of Arthroplasty. 1999	175
82	Jenny J.-Y., Clemens U., Kohler S., Kiefer H., Konermann W., Miehlke R.K. Consistency of implantation of a total knee arthroplasty with a non-image-based navigation system: A case-control study of 235 cases compared with 235 conventionally implanted prostheses. Journal of Arthroplasty. 2005	175
83	Taylor S.J.G., Walker P.S., Perry J.S., Cannon S.R., Woledge R. The forces in the distal femur and the knee during walking and other activities measured by telemetry. Journal of Arthroplasty. 1998	174
84	Peters C.L., Shirley B., Erickson J. The Effect of a New Multimodal Perioperative Anesthetic Regimen on Postoperative Pain, Side Effects, Rehabilitation, and Length of Hospital Stay After Total Joint Arthroplasty. Journal of Arthroplasty. 2006	174
85	Pugely A.J., Callaghan J.J., Martin C.T., Cram P., Gao Y. Incidence of and risk factors for 30-day readmission following elective primary total joint arthroplasty: Analysis from the ACS-NSQIP. Journal of Arthroplasty. 2013	174
86	Bryan D., Parvizi J., Austin M., Backe H., Valle C.D., Kolessar D.J., Kreuzer S., Malinzak R., Masri B., McGrory B.J., Mochel D., Yates A. Obesity and total joint arthroplasty. A literature based review. Journal of Arthroplasty. 2013	174
87	Figgie M.P., Sobel M. The results of treatment of supracondylar fracture above total knee arthroplasty. Journal of Arthroplasty. 1990	173
88	Martell J.M., Verner J.J., Incavo S.J. Clinical performance of a highly cross-linked polyethylene at two years in total hip arthroplasty: A randomized prospective trial. Journal of Arthroplasty. 2003	173
89	Nakata K., Nishikawa M., Yamamoto K., Hirota S., Yoshikawa H. A Clinical Comparative Study of the Direct Anterior With Mini-Posterior Approach. Two Consecutive Series. Journal of Arthroplasty. 2009	173
90	Greene K.A., Wilde A.H., Stulberg B.N. Preoperative nutritional status of total joint patients: Relationship to postoperative wound complications. Journal of Arthroplasty. 1991	172
91	Yoshino N., Takai S., Ohtsuki Y., Hirasawa Y. Computed tomography measurement of the surgical and clinical transepicondylar axis of the distal femur in osteoarthritic knees. Journal of Arthroplasty. 2001	172
92	Ladon D., Doherty A., Newson R., Turner J., Bhamra M., Case C.P. Changes in metal levels and chromosome aberrations in the peripheral blood of patients after metal-on-metal hip arthroplasty. Journal of Arthroplasty. 2004	172
93	Engh Jr. C.A., Stepniewski A.S., Ginn S.D., Beykirch S.E., Sychterz-Terefenko C.J., Hopper Jr. R.H., Engh C.A. A Randomized Prospective Evaluation of Outcomes After Total Hip Arthroplasty Using Cross-linked Marathon and Non-cross-linked Enduron Polyethylene Liners. Journal of Arthroplasty. 2006	172
94	Noble J.W., Moore C.A., Liu N. The Value of Patient-Matched Instrumentation in Total Knee Arthroplasty. Journal of Arthroplasty. 2012	172
95	Lavernia C.J., Guzman J.F. Relationship of surgical volume to short-term mortality, morbidity, and hospital charges in arthroplasty. The Journal of Arthroplasty. 1995	170
96	Healy W.L., Wasilewski S.A., Takei R., Oberlander M. Patellofemoral complications following total knee arthroplasty. Correlation with implant design and patient risk factors. The Journal of Arthroplasty. 1995	170
97	Mancuso C.A., Ranawat C.S., Esdaile J.M., Johanson N.A., Charlson M.E. Indications for total hip and total knee arthroplasties: Results of orthopaedic surveys. Journal of Arthroplasty. 1996	170
98	Weeden S.H., Paprosky W.G. Minimal 11-year follow-up of extensively porous-coated stems in femoral revision total hip arthroplasty. Journal of Arthroplasty. 2002	170
99	Kim S., Losina E., Solomon D.H., Wright J., Katz J.N. Effectiveness of clinical pathways for total knee and total hip arthroplasty: Literature review. Journal of Arthroplasty. 2003	169
100	Pulido L., Parvizi J., Macgibeny M., Sharkey P.F., Purtill J.J., Rothman R.H., Hozack W.J. In Hospital Complications After Total Joint Arthroplasty. Journal of Arthroplasty. 2008	169

**Figure 2:**
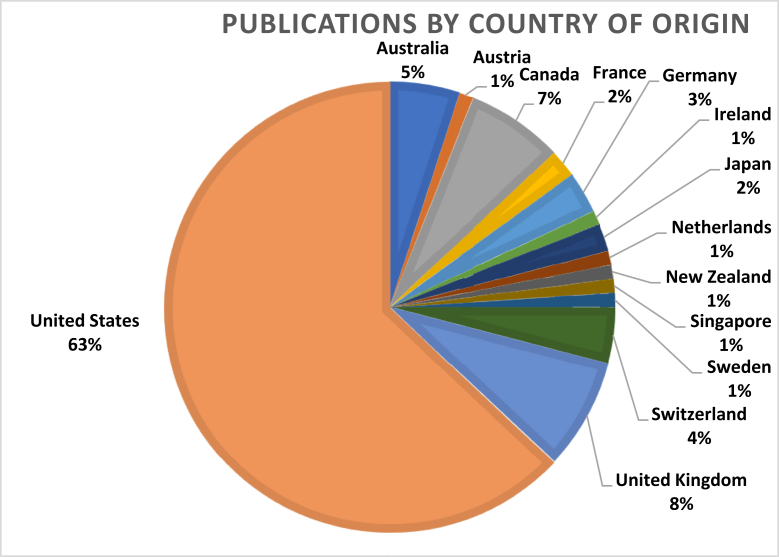
Top 100 articles in the *Journal of Arthroplasty* by country.

Fourteen countries contributed to the top 100 articles. The USA had the greatest number of contributions with 63 articles. The United Kingdom (UK), Canada, and Australia followed with 8, 7, and 5 contributions, respectively. All other countries had less than 4 contributions (Fig. 2).

**Figure 3:**
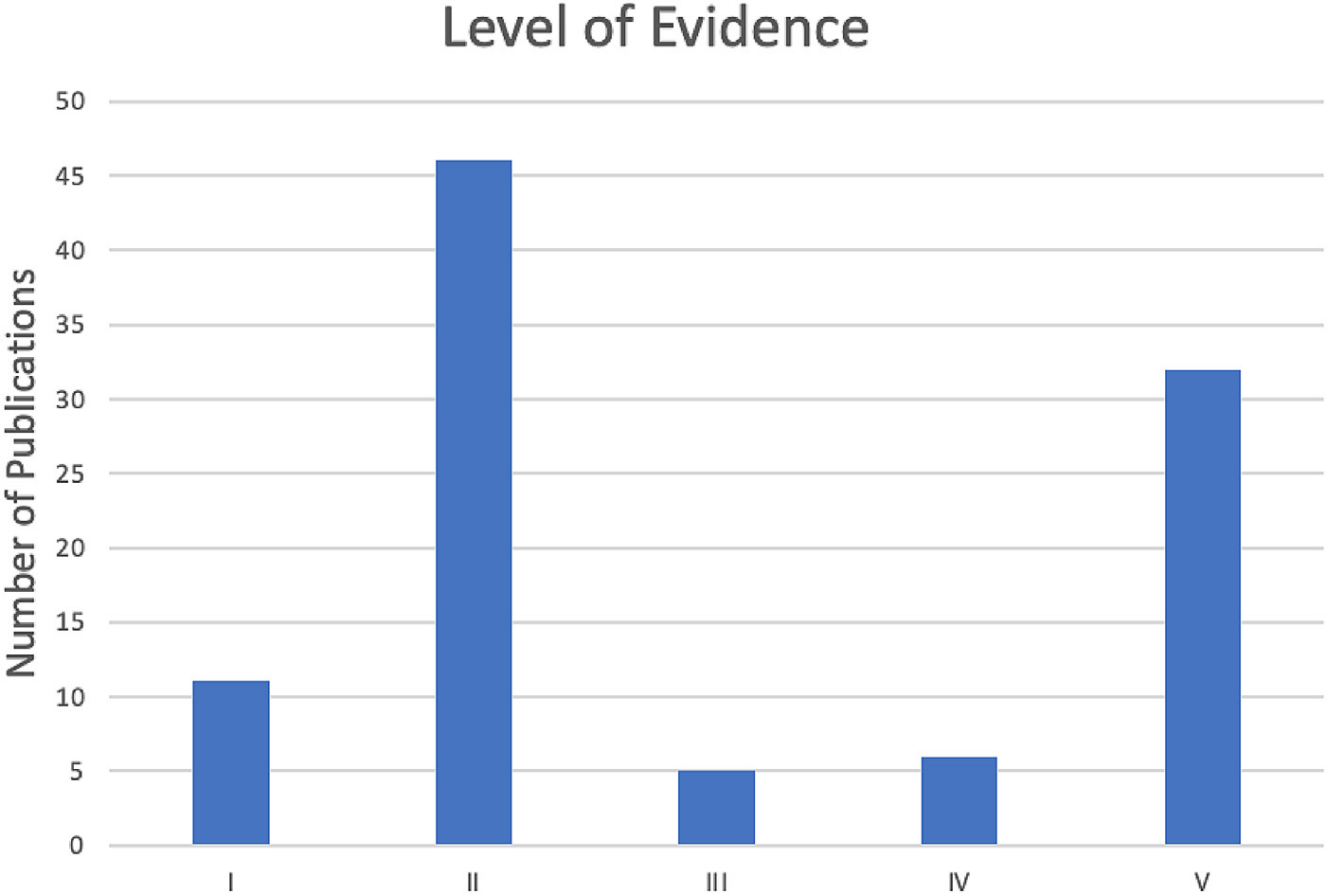
Top 100 articles in the *Journal of Arthroplasty* by level of evidence.

The most recurring LOE was II (n = 46 articles) and V (n = 32 articles). The remaining number of publications at each LOE was as follows: I (n = 11 articles), III (n = 5 articles), and IV (n = 6 articles) (Fig. 3).

**Figure 4:**
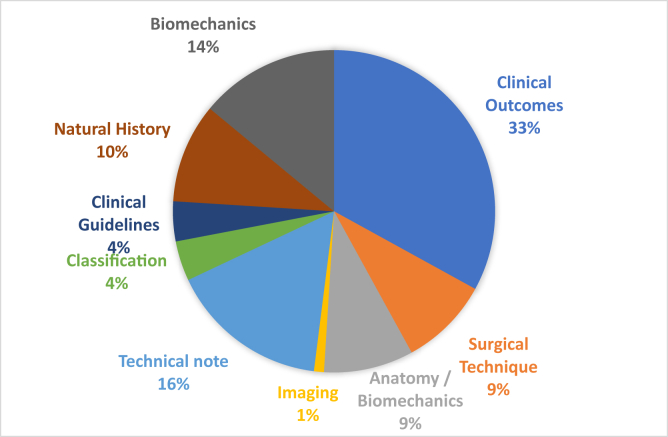
Top 100 articles in the *Journal of Arthroplasty* by type of article.

**Figure 5:**
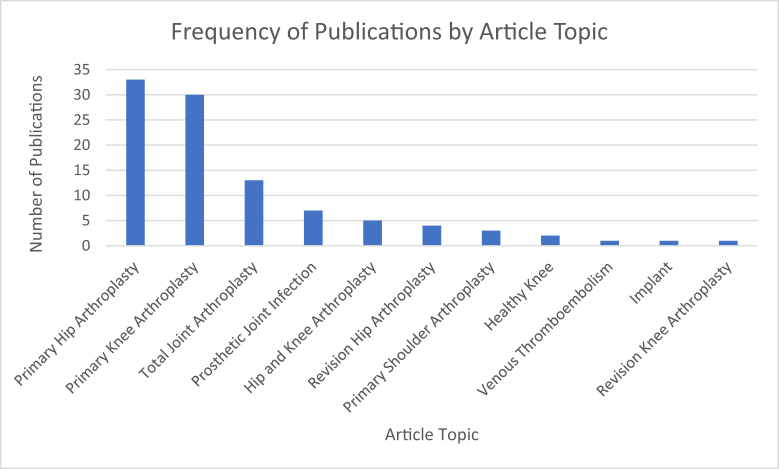
Frequency of publications by article topic.

Articles were classified into 9 different article types. The most frequent article type was clinical outcomes (n = 33 articles), followed by technical note (n = 16 articles) and biomechanics (n = 14 articles). All other articles types have 10 or less publications (Fig. 4). Articles were classified into 11 different topics. The most frequent article topic was primary hip arthroplasty (n = 33 articles), followed by primary knee arthroplasty (n = 30 articles), TJA (n = 13 articles), and prosthetic joint infection (n = 7 articles) (Fig. 5).

## Discussion

This bibliometric analysis highlighted the most influential articles published by the *JOA*. The 100 articles were comprehensively analyzed across categories of publication, LOE, country of origin, authors, and frequency across decade.

Since its establishment in 1991, the American Association of Hip and Knee Surgeons has brought the most up-to-date information on a wide array of scientific topics to its members. Through its original, peer-reviewed articles, the *JOA* has been on the forefront of publishing high-impact medical literature. Previous bibliometric analyses have highlighted the impact that the contributions from this journal have had in the most influential THA and unicompartmental knee arthroplasty research [7,8]. A recent study analyzing authorship trends in the *JOA* found that over time there has been a significant increase in last authors with an MD/PhD and MD/MBA, increases in contribution from international authors, and increases in the mean number of authors per article [9]. The present study highlights the most influential articles published by this journal that have further developed the advancements in arthroplasty research.

Notably, 57% of the publications had an LOE of I or II which is slightly higher relative to other orthopaedic journals. In 2005, Obremskey et al. evaluated the LOE in various orthopaedic journals and found 32% of articles to have an LOE of I or II [10]. In the current study, 11% of the articles had level I evidence while 46% had level II evidence. Over the past 20 years, there has been a significant increase in the number of articles with LOE of I and II possibly due to the increasing emphasis on publication quality in orthopaedics [11].

Additionally, through categorization by article type, we were able to analyze patterns in the top 100 most cited arthroplasty articles. Specifically, clinical outcomes of various interventions comprised about one-third of this list (33%), followed by studies of technical note (16%) and biomechanical studies (14%). Other similar studies have found that clinical outcomes of arthroplasty dominate the orthopaedic literature with respect to citation frequency [3,8].

The most cited article was published by Kurtz et al. in 2012, and it emphasized the financial implications of periprosthetic joint infections. The research team projected that with an increasing demand for joint arthroplasty, cost of infected revisions to US hospitals may exceed $1.62 billion by 2020 [12]. The *JOA* recently published an article with similar evidence pointing toward increasing cost projects primarily due to increases in the total number of these procedures being performed [13]. The third most cited article also came from the work of Dr. Kurtz. This article focused on infection burden for hip and knee arthroplasty [14]. It was published in 2008, during the decade which has had the greatest number of publications on our list. This further exemplifies the academic growth in arthroplasty research during this time span.

The second most cited article followed 147 patients undergoing acetabular component revision and classified their acetabular defects. Their paper stated that by adhering to the used classification system and utilizing the appropriate surgical technique, acceptable and predictable results of acetabular revision can be expected [15]. While most publications in this analysis focused on primary hip or knee arthroplasty, there has been increasing recent research in revision arthroplasty. Specifically with revision THA, there has been a focus on clinical outcomes with most papers having an LOE of II [16]. However, in this analysis, the authors did not find trends changing over time.

Furthermore, the USA is the country of origin for the majority of the articles in this list (63%), followed by the UK (8%) and Canada (7%). This follows the major trend in bibliometric studies, with America contributing the most to medical journals, especially in orthopaedic journals. However, a 2013 study evaluating knee arthroplasty and soft-tissue surgery reported that the USA had declined in publishing over the past 16 years, while the UK and Japan became more prolific in publishing [17]. Nonetheless, the USA continues to contribute the most to top 100 lists in terms of citation frequency [8]. We can attribute this to most renowned medical journals originating in the US, publishing in the English language, and more funding opportunities [18].

There were several limitations of this study. A bibliometric analysis that uses total citation count to rank publications naturally presents bias toward older articles because there has been a longer time period for these articles to accrue cumulative citations. Article classification and LOE were assigned by author review, which involves subjective interpretation despite the use of standard LOE guidelines. The country of origin analysis may not account for the potential multinational collaboration. Outside of Scopus indexing and updates reporting, it cannot be independently verified that all of the most recent *JOA* articles are indexed within the Scopus database.

## Conclusions

Using citation analysis, the most influential articles in the *JOA* were comprehensively and objectively analyzed. The most popular fields of research involved clinical outcomes (33%) and technical note (16%), both of which increase an article’s likelihood of being highly cited. Knowledge of the most influential articles in the *JOA* allows for appreciation of current and potential future areas of literature regarding diagnosis, management, and outcome of a patient undergoing arthroplasty.

## Conflict of interest

Michele D’Apuzzo is a paid consultant at Zimmer Biomet and is a board member of the Florida Orthopedic Society and American Academy of Orthopedic Surgery; all other authors declare no potential conflicts of interest.

For full disclosure statements refer to https://doi.org/10.1016/j.artd.2019.12.004.

## Informed patient consent

The author(s) confirm that informed consent has been obtained from the involved patient(s) or if appropriate from the parent, guardian, power of attorney of the involved patient(s); and, they have given approval for this information to be published in this article.

## Appendix A Supplementary data

Conflict of Interest Statement for Constantinescu

Conflict of Interest Statement for Luxenburg

Conflict of Interest Statement for St. Louis

Conflict of Interest Statement for Bondar

Conflict of Interest Statement for D’Apuzzo

Conflict of Interest Statement for Sudah
